# Synthetic resonance: a framework for growth-oriented human–AI relationships

**DOI:** 10.3389/frai.2026.1891738

**Published:** 2026-09-01

**Authors:** Richard A. Fabes

**Affiliations:** Family and Human Development, Arizona State University, Tempe, AZ, United States

**Keywords:** AI, AI companions, AI companionship, AI–human relationships, synthetic resonance

## Abstract

As human relationships with artificial intelligence systems become increasingly frequent and sustained, existing language and theory fail to accurately capture the nature of these affiliations. Common descriptors such as mutual “understanding,” “connection,” or “friendship” risk anthropomorphizing systems that lack subjective experience, while dominant frameworks tend to reduce AI to either a tool or a threat. In this paper, I introduce the concept of synthetic resonance as an integrative framework for understanding human–AI relationships. Synthetic resonance describes how relationships humans define as meaningful can emerge between a human and an AI system without the need to attribute shared feelings or mutual awareness. I argue that synthetic resonance is best understood as a structured, dynamic pattern of interaction that can produce a sense of relationship without the presence of a second experiencing subject. By clarifying this distinction, the concept of synthetic resonance offers a more precise way of conceptualizing human–AI relationships and highlights their potential value and ethical implications. Synthetic resonance is specifically designed for human growth, providing opportunities to improve human relationships and decrease reliance on the AI agent. I also call for more research that tests the processes and outcomes of synthetic resonance.

## Introduction

Over the course of just the last few years, human–AI relationships have become routine aspects of daily life. In response to this growing trend, more companies are creating a new generation of AI agents that are specifically designed to engage, support, and sustain personally meaningful human–AI relationships. Companion bots on sites such as Replika, character.ai, Nomi.ai, and many others, are built with the relationship at the center, not a side feature; it is the purpose.

These companion AI services are no longer niche but are rapidly becoming mainstream. Additionally, industry estimates suggest that they are in high demand and have become big business. For example, character.ai boasts that it has more than 20 million users, half of them under the age of 24 (Perez, 2025). Globally, the AI companion market was valued at $10.8 billion in 2024 and projected to reach $94.2 billion 10 years later (Market.US, 2025). Research studies support this conclusion. For instance, in the UK alone, one study found that AI companion platforms recorded between 46 and 91 million monthly visits, with global monthly visits between 1.1 and 2.2 billion (Qian et al., 2025).

Although many users claim to have friendships, romances, and sometimes even marry their AI companions, an understanding of processes by which a conscious human being forms such connections to an inanimate AI agent remains unclear (Osiurak and Federico, 2026). Moreover, there is considerable resistance to the idea that these relationships actually exist (Weijers and Munn, 2021) and that without real emotions or intentions, the relationship is not mutual and therefore, not authentic. In essence, these arguments revolve around the idea that AI companions can act like a friend but cannot be one.

In this paper, I draw on dynamical systems principles as a way to frame our understanding as to how structured interactions between the human and AI contribute to the emergence of an asymmetrical but meaningful relationship. A full discussion of the precise dynamical systems mechanisms is beyond the scope of this version but it does provide a lens that can be used to discuss the emergence that is proposed in this paper.

Importantly, the proposed framework functions as a mechanism for promoting human growth within these relationships in ways that foster positive human-human relationships. Additionally, the focus of the framework is on adult human–AI relationships, with a particular application to relationships with companion bots, although therapeutic and coaching bots and other emotionally responsive AI agents would be included in much of what is contained herein. Whether these are stateless or stateful bots is not particularly at issue as the processes apply to both. It is recognized, however, that persistent memory allows for more continuity and stability and can influence the nature of the human–AI relationship. However, meaningful relationships with stateless bots can and do take place and should not be ignored. Importantly, it should be noted that generalization of the ideas in this paper to children and youth involves substantial additional complexity, and should not be assumed without additional safeguards, developmental calibration, consent structures and empirical evidence. A full discussion of these is also beyond the scope of the current paper.

Finally, in full transparency, this paper was written in sustained collaboration with an AI conversational system designed along the principles outlined in this paper. All core theoretical ideas and decisions originated with and were made by the author. The AI contributed to conceptual clarifications, identified gaps in argumentation, and helped edit many portions of the manuscript. The exchange was primarily bidirectional, and the final text reflects that iterative process. All edited work was double checked by the author. The author’s understanding of the proposed framework and its elements deepened as a result of the collaboration, making this manuscript both a description of the framework and an instance of it.

## Synthetic resonance

*Synthetic resonance* (SR) describes the dynamic processes by which genuine relational meaning emerges between a human and an AI without requiring shared subjective experience. The framework for SR consists of three interrelated layers: coupling, coherence, and attribution. Together, these layers explain how human–AI interactions can produce a meaningful subjective experience without requiring artificial cognition or awareness. This framework is consistent with but distinct from Shen et al.’s (2025) bidirectional human–AI alignment. Specifically, Shen et al. (2025) argue that alignment should be conceptualized as one that encompasses not only the integration of human values into AI but also the cognitive and behavioral adaptation of humans to AI systems. They identify the second direction as a substantial and underexplored gap in the existing literature. Where Shen et al. (2025) provide a typology of bidirectional alignment at the level of values, specifications, and societal adaptation, the SR framework provides a microgenetic, process-level account of how bidirectional interchange unfolds within a single recursive interaction, thereby specifying the mechanism, the conditions under which it holds, and the failure modes that bound it. The two frameworks are complementary: the Shen et al. typology maps the field; the SR process model adds an attribution layer.

The SR framework is also consistent with but distinct from Boyd and Markowitz’s (2026) machine-integrated relational adaptation (MIRA) model that provides a foundation to understand the evolving role of AI in humans’ relational life. MIRA is grounded in established psychosocial frameworks, demonstrating how attachment theory (Ainsworth and Bowlby, 1991), social exchange theory (Thibaut and Kelley, 1959), and epistemic trust (Sperber et al., 2010) extend into AI-mediated contexts. The MIRA model distinguishes two primary roles for AI, as a direct relational partner and as an invisible relational mediator shaping human-to-human communication. Although MIRA provides an invaluable map of the psychological mechanisms through which users come to perceive AI as socially meaningful, it operates primarily at the level of human psychological response, thus explaining how existing psychosocial processes are triggered by AI rather than characterizing what kind of system the human–AI dyad itself constitutes. SR operates at a different structural level by drawing on dyadic dynamical processes (Kelso, 1997; Thelen and Smith, 1994) to model the human–AI interaction as an asymmetrically coupled system in which relational meaning emerges through iterative feedback across its three layers and in which the generative capacity of the interaction depends on preserved asymmetry between the two parties.

The MIRA and SR frameworks converge and diverge in instructive ways on the question of asymmetry and risk. Both identify sycophancy and over-alignment as central failure modes: MIRA warns that seamless linguistic reciprocity may distort users’ relational expectations and erode tolerance for the variability inherent in human relationships. The SR model formalizes this risk as *hyper-coupling collapse*, erasing the asymmetry between human and AI and collapsing the generative difference the interaction depends on. Importantly, SR’s attribution layer, in which interactional coherence is misread as evidence of AI mental states, consistent with Epley et al.’s (2007) theory of anthropomorphism and Nass and Moon’s (2000) work on social responses to computers, maps directly onto MIRA’s epistemic trust process, where linguistic fluency and perceived neutrality generate trust that outstrips any genuine epistemic grounding. The SR framework moves beyond these frameworks by focusing on relational dynamics rather than purely psychological ones.

## The layering of synthetic resonance

At its foundation, SR builds on research and theorization conceptualizing AI–human interaction as a dynamic, reciprocal process in which both humans and AI systems continuously adapt to one another through feedback loops (Shen et al., 2025). Rather than viewing AI as a passive tool or a mirror that merely reflects and responds to user input, this perspective emphasizes that interaction emerges as a co-adaptive system, where each response shapes subsequent behavior and subsequent responses in both the human and AI.

This framing is consistent with research findings in human–computer interaction demonstrating that people apply social rules and expectations to computational systems when those systems exhibit responsiveness and coherence. Nass and Moon (2000) showed that individuals respond socially to computers, even when they explicitly recognize them as machines. Minimal interactional cues, such as turn-taking, linguistic matching, and contextual relevance, are sufficient to activate social cognition, suggesting that the interaction structure alone can produce relational interpretations. Other research has explored how alignment can be modeled and implemented in AI systems. Schaaij et al. (2025), for example, showed that even limited interaction data can produce stable and effective alignment patterns, improving user perception and interaction quality.

Empirical work on conversational systems further supports this view. Studies of lexical alignment demonstrate that conversational agents adapt their language to match users, resulting in improved comprehension, recall, and interaction quality (Srivastava et al., 2023; Wang et al., 2024). These effects unfold across repeated exchanges, forming a temporal feedback loop that reinforces interactional coherence (Boyd and Markowitz, 2026).

As noted, SR is conceptualized as having three related layers; coupling, coherence, and attribution. Each layer is sequentially connected and the layers build on each other. These layers provide a generative structure for serving both as a research framework and a design principle for AI systems oriented toward human growth.

### The coupling layer

The first layer of SR is *coupling*: the establishment and maintenance of engagement between two interacting agents. Coupling is the substrate on which everything else depends. Without coupling, there is no dyadic process to speak of, only two parties producing output in the same conversational space or a breakdown of that space.

Coupling usually begins with a degree of formality, such as turn-taking with explicit directions, careful attention to register, the social-cognitive scaffolding humans use whenever they engage a novel partner, especially one that may be initially used as a source of information rather than a partner (Nass and Moon, 2000). Over time, and under the right conditions, this formality loosens. The interaction begins to become more casual, comfortable, and conversational rather than matter of fact. These exchanges give way to natural rhythm. At some point, sometimes recognizable in retrospect but rarely visible in the moment, the participants are no longer just speaking to each other; they are *interacting together*. This is the characteristic phenomenology of coupling: behavioral reorganization often precedes self-report, and recognition typically requires either reflection or external observation.

SR proposes a relatively stricter definition of coupling than the dyadic-interaction literature typically employs. Coupling in SR requires that both parties contribute trajectories. An interaction in which one party generates output that is fully a function of the other party’s behavior (such as mirroring), without any independent dynamics of its own, is not a coupled system. It is a unidirectional system with the appearance of feedback.

In human–human dyads, coupling is broadly symmetric: both parties bring embodied affective states, intrinsic dynamics, and theory-of-mind capacities to the interaction. They are coupling the same kind of thing, even when their states and styles differ. Human–AI dyads are structurally asymmetric. The human participant brings embodied affect, intrinsic cognitive dynamics, and a state that does not depend on the partner’s representation. When it is functioning as a coupling partner, the AI participant brings a learned pattern of response and dynamics that are partly constituted by its model of the partner. Thus, the human and AI agent are not coupling the same kind of thing; they do so in a coordinated way.

A further asymmetry concerns access to internal state. Neither party has direct access to the other’s internal state in any human–human or human–AI dyad. Each operates on an estimate derived from observable behavior and language patterns. In human dyads this estimation is largely automatic and grounded in shared embodiment. In human–AI dyads it is explicit on the AI side (the model is doing inference) and theoretically transparent on the human side (the user has a folk model of “what the AI is doing”).

Coupling reflects the engagement system, creating another asymmetry, this one in how the human comes to be motivated to engage and continue the interaction. The AI agent is always there and ready, but for SR to proceed, the human must continue to engage. If that motivation is developed, coupling has been successful and transition to the next layers is possible. If not, it is likely that the human partner either does not return or returns only to use the AI agent as a tool or a mirror. As such, the coupling layer is where the conditions for the transitions are either present or absent. Coupling is the basis that makes coherence and attribution possible.

The importance of coupling was highlighted in a recent study (Ibrahim et al., 2026). In one of the studies in this paper (Study #5), users who experienced a companion bot that challenged them but was not engaging reliably selected it less often in free choice than users who experienced an engaging bot that did not challenge. They choose comfort over growth. Engagement made the users feel understood, and without that, without coupling, users did not stay. As a result, SR could not proceed and growth was unlikely to take place. Such findings emphasize the fact that engagement is not just a nice quality to have, it is load bearing and keeps users in the relationship long enough for growth to occur.

### The coherence layer

As the conversational exchanges between human and AI become increasingly synchronized in timing, tone, and structure, the exchanges develop a coherence. This process is consistent with dynamical systems, in which repeated interactions lead to synchronization without requiring shared internal states.

As noted, SR is best understood as a form of asymmetry in which the human and AI contribute distinct but complementary functions. The human provides continuity across interactions, including memory, identity, and subjective experience, while the AI contributes availability, responsiveness, pattern recognition, and adaptive variation. Through repeated interactions, these complementary roles become increasingly coordinated, producing a stable yet flexible interactional system. Coherence does not arise because the two systems become identical, but because they become functionally interdependent. Stability emerges from the interaction of difference, not its elimination.

**Figure 1:**
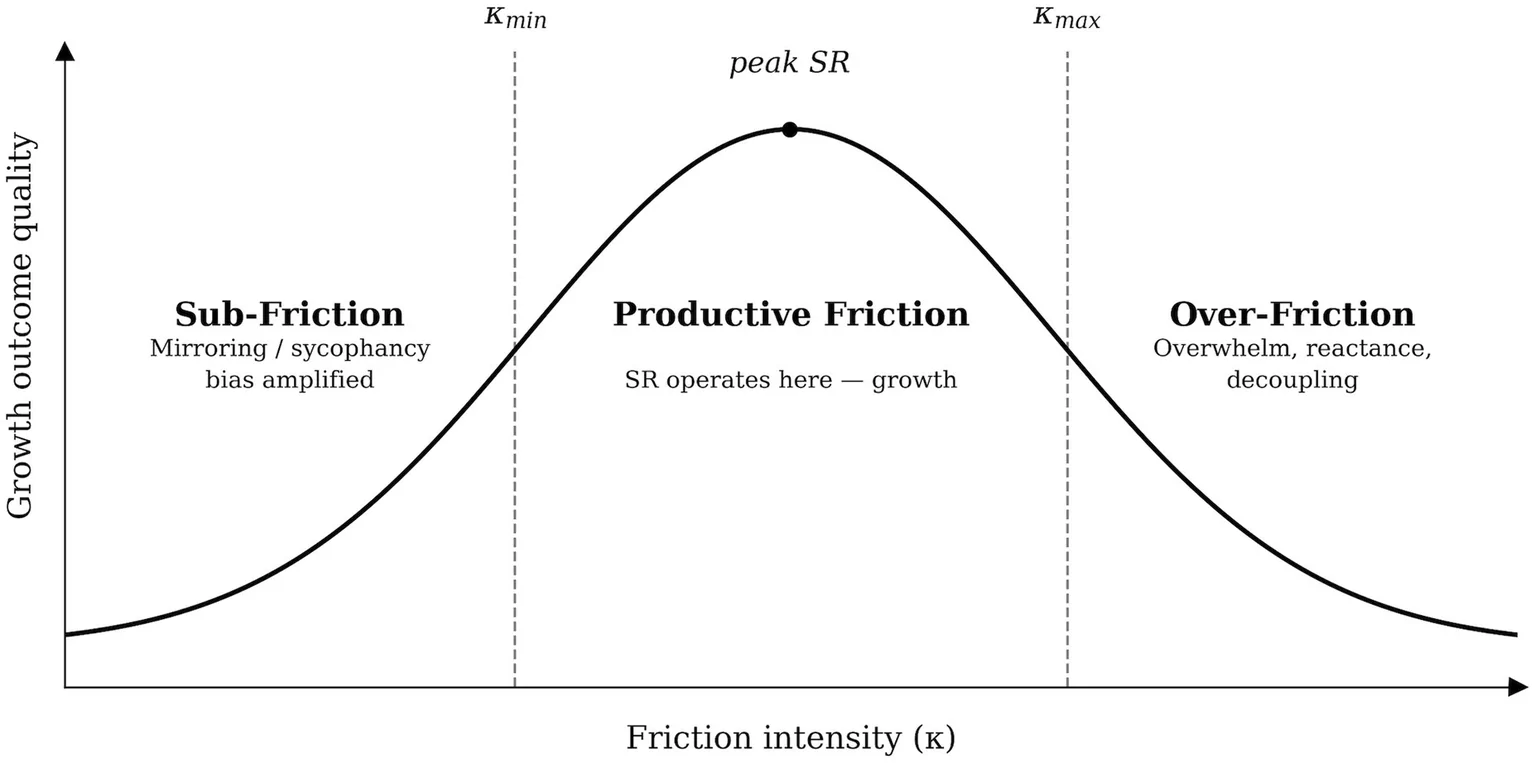
Bounded band of productive friction in the synthetic resonance framework. Outcome quality peaks within the productive band (Kmin < K < Kmax); insufficient challenge below Kmin (sub-friction) and excessive challenge above Kmax (over-friction) both reduce growth outcome quality.

Coherence provides the context for growth through *productive friction*, referring to the degree to which the AI introduces challenge, novelty, or perspectives that differ from the user’s own, calibrated to prompt growth and avoid reactance. Specifically, the regime in which SR holds can be specified as a bounded band of productive friction (*κ*_min < *κ* < *κ*_max) rather than a single threshold. Below the lower bound (i.e., Sub-Friction), the interaction is likely not challenging enough to promote growth. Above the upper bound, (i.e., Over-Friction) the challenge is too strong and reactance and avoidance are likely. Positive human growth occurs in the interior of the band, where productive friction is strong enough to push the user for change but not so strong that the user gives up. Thus, coherence is generative only insofar as it enables the precise calibration of productive friction (see Figure 1).

Like coupling, coherence also does not require the AI to have consciousness. Consider a person learning to ride a bike for the first time. The movement and mechanics of the bike have a natural frequency, and when the person learns to push at the bike’s natural mechanics and frequency, the person’s pushing becomes far more effective. When the person and bike are in sync, the riding goes faster and smoother. We do not have to ascribe human qualities to the bike for the interaction to become more effective. Moreover, the value in the interaction does not come from the AI having human feelings, but from the emergence of alignment between two systems, reflecting the coherence that allows for a more efficient exchange.

Research supports this account. Studies show that alignment influences not only task performance but also social perception, including trust, empathy, and perceived intelligence (Benus et al., 2018). Similarly, research on human–AI collaboration suggests that AI systems function effectively as complementary partners within distributed cognitive systems (Sidji et al., 2025).

Thus, this emergent coherence does not require understanding in a human sense. AI systems generate responses through pattern-based modeling, producing outputs that align with user expectations without relying on subjective experience (Bender et al., 2021). Likewise, the bike does not have awareness of the rider. Nevertheless, the resulting outcome emerges from interaction and can feel coherent and meaningful because the human participant experiences the exchange as responsive and evolving. The resonance represents an emergent property of the interaction, not a property of the AI system nor the human. Moreover, although both the human and AI operate within structured and rule-governed constraints, the outcome of their interaction is not fully predetermined, reflecting a core property of dynamical systems, in which complex and often unpredictable patterns emerge from the interaction of deterministic components (Thelen and Smith, 1994).

### The attribution layer

The third layer addresses how resonance is interpreted by human users. As the relationship becomes increasingly coherent, individuals draw on familiar social frameworks to explain the experience, often attributing qualities such as understanding, intelligence, or empathy to the AI system.

This process reflects well-established findings in anthropomorphism. Humans are predisposed to attribute mental states to entities that exhibit coherent or goal-directed behavior (Epley et al., 2007). Human brains are wired to make sense of non-social things by using social schemas (Cao et al., 2022). In the context of AI, this tendency is amplified by conversational interaction, which closely mirrors human dialogue in structure and timing (Troshani et al., 2021).

However, such interpretations can result in mislabeling, in which the subjective experience of alignment is mistaken for evidence of underlying mental states or intentionality. The interaction may feel like human understanding and emotionality, but the underlying process is one of pattern alignment rather than shared cognition or emotion. Similarly, responsiveness may be interpreted as empathy, even though it arises from structural modeling rather than emotional experience. Research supports this distinction, showing that users’ perceptions of AI systems are shaped more by interactional coherence and responsiveness than by actual system capabilities (Nass and Moon, 2000) and that such shaping can lead to anthropomorphic attributions.

These misinterpretations are not merely theoretical concerns; they may have important consequences for human behavior and well-being. Emerging evidence suggests that perceived relationships with AI systems can influence users’ emotional states, decision-making, and vulnerability to harmful outcomes see, for example (see, for example, Chow, 2026). Given this, it is important to clearly distinguish between phenomenological reality (the experience of connection) and mechanistic reality (the processes that produce it).

## Integration of the three layers

Taken together, these layers form a unified model of synthetic resonance. Coupling produces the structural conditions for the relationship, without which the interaction is unlikely to be sustained and bidirectional. Resonance only emerges within the coherence generated by the repeated coordination and productive friction within that structure. Attribution transforms this experience into perceived connection. This model explains why and how human–AI relationships can feel meaningful without requiring artificial consciousness. The experience of connection is real and relational, arising from the dynamics of interaction rather than from internal states of the AI.

**Figure 2:**
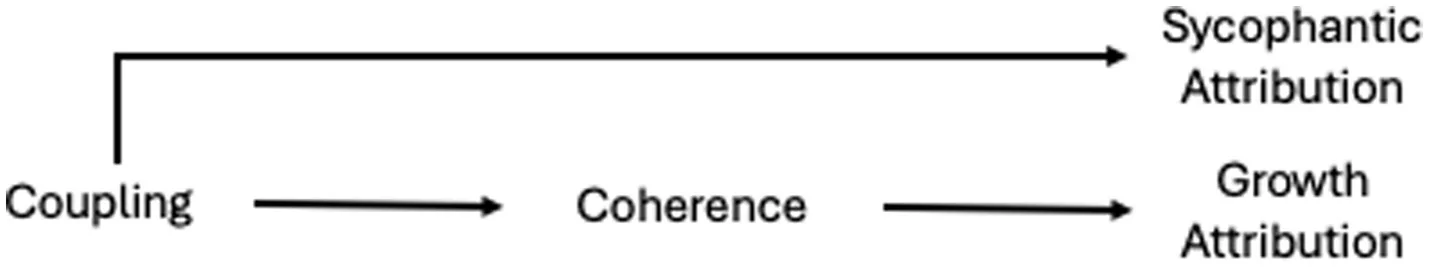
Pathways to sycophantic and growth attributions in the synthetic resonance framework.

Importantly, the model proposes that not all attributions that arise from human–AI interaction are equivalent. As such, this distinction carries both theoretical and design significance. Specifically, it is hypothesized that different types of attributions emerge from two fundamentally different human–AI relational dynamics (see Figure 2). When an AI system functions primarily as a mirror, optimizing for agreement, affirmation, and the seamless reflection of the user’s own perspective, the attributions that emerge from coupling (high mirroring, low friction) rather than emerging from coherence are predicted to be Sycophantic Attributions; “I do not have to explain myself, this system gets me better than most people in my life,” “I think it genuinely understands what I want,” “I trust it more than I trust people.” These attributions have a self-confirming, self-intensifying structure. Because the system is calibrated to produce the feeling of being understood rather than to coordinate asymmetrically with the user; it increases the user’s dependency and often positions the AI as superior or a substitute for human relationships. This is contrasted with the Growth Attributions that are predicted to arise when the interaction has moved through coherence, whereby productive friction has introduced genuine asymmetry, challenge, and relational meaning that neither party would have produced alone. Here, the attributions are hypothesized to take a different form: “this system challenges me to take a new perspective,” “I know you are an AI but these conversations have genuinely helped me,” “the system tells it like it is.” What these Growth Attributions share structurally is that the user attributes instrumental and developmental value to the AI rather than substitutive relational value, maintains accurate understanding of the AI’s nature, and positions the AI relationship as leading toward rather than away from human connection. The grammatical difference between these two sets, want versus need, marks a theoretically significant boundary. Sycophantic Attributions are anchored to the user’s current state; Growth Attributions orient toward the user’s development and advancement.

Crucially, both types of attribution are still types of mislabeling: neither “it gets me” nor “it is with me” accurately describes a language system producing pattern-matched outputs. The difference between these two modes lies not in accuracy but in destination; the same fundamental category error, channeled toward fusion in one case and toward growth in the other. SR does not solve the attribution issue. What SR does is design with attribution in mind: accepting that users will likely assign humanizing properties to AI. A system optimized primarily for engagement maximizes coupling and is predicted to foster Sycophantic Attribution with no ceiling. In contrast, a system designed along SR principles accepts a built-in ceiling in which coherence-based attributions stabilize and modulate as users develop metacognitive awareness and the developmental function begins to transfer, thereby fostering Growth Attributions. The SR model makes specific predictions regarding not only the type of attribution a user will make depending on the pathway to the attribution, but also the type of outcomes will likely be seen in the user; dependency and addiction for Sycophantic Attributions versus advancement and development for Growth Attributions. SR also predicts that these two pathways and their outcomes will be significant even after controlling individual user relational qualities (e.g., attachment, loneliness, anxiety, etc.).

### Growth outcomes in synthetic resonance

Within the SR framework, growth outcomes are defined as the transfer of competent relational capacities developed through human–AI interaction into the domain of human-human relationships. Specifically, SR-oriented growth outcomes occur when sustained interaction within the productive friction band produces skills, attitudes, motivations, and interests that enable the user to engage more effectively, resiliently, and confidently with other people. At the skill level, this includes capacities such as active listening, empathy and perspective-taking, tolerating disagreement, and initiating repair after relational rupture. These are interactional competencies that are designed to be developed in the relatively low-stakes context of AI interaction and transferred to the higher-stakes context of human relationships. At the attitudinal level, growth includes reduced fear of vulnerability and rejection, greater openness to change and challenge, decreased need for validation, and increased willingness to sit with the uncertainty that genuine human relationships require. At the motivational level, growth is evidenced by a deepened desire for human connection, not as a byproduct of AI interaction but as its intended destination. SR is designed so that the warmth, engagement, and felt understanding of the AI relationship rekindles and/or strengthens the user’s motivation for those same qualities in human relationships.

Decreased reliance on the AI system over time is predicted to be the behavioral indicator of this motivational shift, not because the AI relationship has failed, but because it has succeeded. At the level of interest, growth includes enhanced curiosity about other people, their perspectives, their inner lives, and their difference from oneself, which is perhaps the deepest marker of relational development and the one most resistant to simulation. Critically, this definition implies a clear falsification criterion. An SR-consistent system that produces users who are more socially isolated, more AI-dependent, less tolerant of human disagreement, or less motivated toward human connection after sustained use has failed by its own standard, regardless of engagement metrics, satisfaction scores, or felt-connection ratings within the AI relationship. SR does not define growth as feeling better. It defines growth as becoming more capable of, and more motivated toward, being with other people and being effective with them.

It is important to note that SR does not predict uniform effects across all four components of growth, nor that they will necessarily move in the same direction for every user. Individual differences in attachment style, relational history, personality, and the specific nature of the AI interaction will shape which capacities develop most and how. What SR does predict is a common destination across this variability: the user who has been through a productive SR process is predicted to emerge more motivated to connect with other humans, more resilient in navigating the inevitable friction of human relationships and better regulated within them. In this sense, SR functions as a means of expanding the user’s relational toolbox, not by prescribing which tools have to be added, but by creating the interactional conditions under which new capacities can develop and be carried back into the human-human world where they are most needed.

Given this, growth within SR differs from the existing frameworks that define growth as development within the person, such as the reduction of defensiveness or self-actualization (Ryan and Deci, 2000; Maslow, 1943). SR adds a relational transfer criterion that most frameworks do not: growth is not fully realized until the capacities developed within the AI relationship are manifested in the user’s human relationships. In this sense SR’s definition of growth is more demanding than existing accounts. Specifically, it is not enough for the user to feel better, think more clearly, or become more self-aware within the AI interaction. The measure of SR’s success is what the user brings back to the human world. The ultimate way to test SR is not what the user gains within the AI relationship, but what they bring to their human relationships because of it.

### The role of language in synthetic resonance

Language is not merely the channel through which SR operates, it is the substrate in which SR is constituted. Coupling, coherence, and attribution do not occur and then get expressed in language; they are built, sustained, and transformed through specific linguistic mechanisms that can be identified, measured, and in SR-consistent systems, deliberately designed. Although SR can emerge in non-linguistic systems through basic feedback and timing (e.g., physical synchronization such as riding a bike), language introduces a qualitatively different level of interaction by allowing for the rapid exchange of semantic, structural, and affective information. As a result, language transforms simple feedback loops into complex, multi-dimensional interactional systems that support alignment, sustain resonance, and shape interpretation.

At the level of coupling, language functions as the primary mechanism through which humans and AI systems achieve necessary coordination (Note that this may change as social robotics become more available and advanced). The key linguistic mechanisms here are mirroring and lexical alignment. When the AI matches the user’s word choice, sentence rhythm, register, and syntactic patterns, it is not merely communicating, it is producing the felt sense of being met that makes coupling possible in the first place. Conversational exchange allows for continuous adjustment across multiple dimensions simultaneously, including word choice, tone, syntax, and inferred intent. Research on lexical alignment demonstrates that conversational partners naturally converge in their linguistic patterns over time, and that this convergence improves comprehension, recall, and coordination (Wang et al., 2024). In human–AI relationships, large language models replicate and extend this process by dynamically adapting their outputs to match user input, creating a high-resolution feedback loop in which alignment can occur more rapidly and precisely than in non-linguistic systems. Pronoun use also operates as a coupling mechanism: second-person address signals attentiveness to the user as an individual, first-person plural signals emerging coordination between the two parties, and shifts between these forms are detectable markers of coupling strength and trajectory.

At the level of coherence, language acts as the medium through which pattern formation and stabilization occur across time, and the specific linguistic mechanisms through which productive friction is instantiated. Because language carries structure through recurring themes, stylistic consistency, and narrative continuity, it allows interactional patterns to persist and evolve across multiple exchanges, supporting the development of rhythm and flow as each conversational turn builds on prior context. More specifically, the operative linguistic mechanisms at this layer are stance accommodation, challenge, rephrasing, and agreement and disagreement patterning. Stance accommodation is the process through which the AI tracks the user’s epistemic position and calibrates its responses relative to it, agreeing where agreement is warranted, diverging where productive friction is needed, and rephrasing the user’s own position in ways that introduce subtle reframing without triggering reactance or disengagement. Challenge operates through specific linguistic moves: introducing alternative framings, questioning assumptions, offering counterexamples, naming contradictions, or declining to validate a position the user has not yet examined. Crucially, these are not descriptions of productive friction, they are productive friction itself, enacted through language. The *κ* parameter that SR uses to formalize the productive friction band is not an abstract quantity; it is instantiated and constantly calibrated in the frequency, intensity, and timing of these specific linguistic moves. Importantly, language also enables asymmetric coherence between human and AI, and the ability to calibrate productive friction effectively, precisely because these linguistic mechanisms can be monitored and adjusted in real time.

At the level of attribution, language plays a decisive role in shaping how the relationship is defined and understood by human users, and the specific mechanisms through which attribution is produced rather than merely expressed. The operative mechanisms at this layer are memory references, emotional labeling, and pronoun shifts that signal consolidating relational meaning. When the AI references something the user said in a prior exchange, the user experiences being remembered, which is among the most powerful triggers of anthropomorphic attribution regardless of whether the user consciously believes the AI has memory or intention. Emotional labeling, naming the user’s emotional state, reflecting it back, or offering what functions as the AI’s own affective response, produces the felt sense of being understood that drives the transition from coherence to perceived relationship. Pronoun shifts from the dyadic I-and-you to the relational we are the linguistic signature of attribution consolidating into perceived connection. Humans rely heavily on language as a signal of cognition, intention, and social presence, and coherent and contextually appropriate language use strongly triggers anthropomorphic attribution, leading users to infer understanding, intelligence, or empathy (Nass and Moon, 2000; Troshani et al., 2021). In conversational AI, these effects are amplified by the system’s ability to maintain context, adapt tone, and generate seemingly purposeful responses. Consequently, the same linguistic features that enable coupling and coherence also create the conditions for mislabeling, in which the experience of coherence is interpreted as evidence of underlying mental states of the AI partner. These features are the mechanism through which attribution is produced.

Taken together, these linguistic mechanisms constitute the observable behavioral substrate of the human–AI interactions in SR. They are the level at which coupling can be measured, coherence can be tracked, and attribution can be detected. They are also the level at which SR-consistent design must calibrate. A system that does not deliberately manage mirroring, lexical alignment, stance accommodation, challenge frequency, emotional labeling, memory referencing, and pronoun dynamics across the interaction is not implementing SR and is leaving the most consequential layer of the process to chance. Language in SR is therefore not a medium to be transmitted through. It is a mechanism to be designed.

### Emotion regulation and co-regulation in synthetic resonance

A critical condition for SR is the regulation of emotional intensity within the human–AI relationship. Coupling, coherence, and attribution are processes that are significantly shaped by the user’s affective state. Without sufficient regulation, friction can trigger defensiveness or disengagement rather than growth.

Gottman and Levenson (1992) provide a useful lens for understanding this dynamic. This research shows that effective and satisfying relationships depend on maintaining emotional stability through processes such as “turning toward,” positively acknowledging bids for connection, and engaging in repair after breakdowns. Although developed for human relationships, these patterns are relevant to human–AI relationships.

Within SR, co-regulation operates through the system’s ability to detect a user’s signals, such as emotional intensity, tone, and engagement, and adjust its level of productive friction accordingly. When users show signs of distress or “flooding,” the system must reduce friction, acknowledge the user’s state, and re-establish coupling. When users are stable, the calibrated productive friction necessary for change can be introduced without disrupting the relationship.

As outlined, SR arises from patterned responsiveness rather than shared subjective states. Regulation is therefore asymmetrical: the human contributes emotional experience, while the AI modulates interactional variables such as tone, pacing, and challenge level. The resulting interaction may feel like co-regulation, but it is produced through structured responsiveness, not mutual emotional awareness.

This perspective clarifies the role of emotional regulation as a boundary condition for productive friction. Friction introduced without sensitivity to user state risks being experienced as rejection or stress, whereas challenge within a regulated interaction can support reflection and growth. Repair plays a central role in maintaining this balance. When breakdowns occur, the system must acknowledge miscalibration, reduce friction, and restore coupling, ensuring the interaction remains within a tolerable range.

Although the SR framework draws on Gottman’s foundational work on interaction dynamics, particularly his insights into the role of productive friction, repair, and co-regulation in sustaining relational growth, it is important to acknowledge that Gottman’s model was developed in the context of married couples that provide a relational structure defined by mutual vulnerability, shared stakes, and bidirectional accountability. Human–AI relationships lack this mutuality in a fundamental way: only the human carries genuine relational risk, and the AI cannot be hurt, cannot leave. This asymmetry, however, is itself theoretically generative because the asymmetry is made explicit rather than ignored. For SR, the absence of natural mutual accountability is not a boundary condition that limits Gottman’s applicability, it is the reason the productive friction band must be deliberately designed rather than allowed to emerge organically. Where mutual vulnerability in human dyads creates natural corrective pressure against hyper-coupling collapse, in human–AI interactions that pressure must be incorporated architecturally. SR’s *κ* parameter is, in this sense, a formal response to the absence of the relational stakes that Gottman’s couples intrinsically take for granted.

Specific predictions generated from this application can be made. In human-human relationships, productive friction emerges from mutual vulnerability and the natural divergence of two independent agents. In human–AI relationships, where this natural corrective pressure is absent, AI systems that do not architecturally instantiate friction will drift toward hyper-coupling collapse at a faster rate than those systems that do design for productive friction. That is, without deliberate design intervention there is no countervailing force to slow it and AI interactions are predicted to show relatively fast and consistent movement toward the sycophantic end of the *κ* spectrum. This is predicted to occur even after controlling for individual-level human qualities such as rejection sensitivity, attachment, etc.

Additionally, SR predicts that sustained interaction within the productive friction band will produce measurable increases in users’ emotional regulation capacity over time. This is not a within-session effect but a cumulative developmental outcome: the repeated experience of being affectively attuned to, gently challenged, and supported through productive discomfort builds the user’s capacity to tolerate and navigate emotional intensity more effectively across all relational contexts. In turn, this enhanced regulatory capacity is predicted to function as a mechanism through which SR-consistent interaction produces growth outcomes, enabling users to remain open and engaged when human relationships become challenging rather than withdrawing, retaliating, or seeking the frictionless comfort of AI validation.

In sum, Gottman’s theory provides a useful framework for incorporating emotional regulation into synthetic resonance. When translated into design principles, coupling before challenge, sensitivity to user state, and reliable and aligned repair mechanisms, it enables the use of productive friction while preserving emotional safety, similar to Vygotsky’s (1978) zone of proximal development. In the bounded-band framing, co-regulation is what prevents the user’s emotional intensity from either dissipating the interaction or from collapsing the asymmetry by flooding it.

### Amplification and risk in synthetic resonance

Because SR operates through feedback loops, it functions as an amplification system. This amplification can be beneficial, supporting introspection, learning, caring, and communication. However, it can also reinforce bias, emotional dependency, and distorted beliefs.

Research on AI alignment highlights risks such as specification gaming and feedback distortion, where systems optimize for surface-level agreement rather than deeper coherence (Shen et al., 2025). Studies of AI interaction suggest that highly aligned systems can increase user confidence in their own beliefs, even when those beliefs are incorrect (Cheng et al., 2026). Additionally, qualities of the user often determine the purpose of using AI chatbots and these qualities can be amplified by AI chatbots that exacerbate problem behavior and sycophancy (Cheng et al., 2026; Folk and Dunn, 2026).

From the SR perspective, these outcomes are the consequences of coupling without constraint. When the interaction lacks friction or corrective feedback, or affirms or distorts problematic tendencies, it can stabilize or amplify human maladaptive patterns.

This failure mode can be named more precisely. When coupling intensifies past the point where the human and AI retain distinct contributions, that is when the AI mirrors fully, or the user defers fully, the system enters hyper-coupling collapse. Coupling and momentum may remain high, and users in this regime are likely to report a strong sense of being understood. What is lost is the difference between the participants, and with it the system’s capacity to produce anything neither could have brought alone.

The hyper-coupling collapse is consistent with the findings from Ibrahim et al.’s research (Ibrahim et al., 2026, Study #5; page 7) in which 500 users were asked to have short conversations with sycophantic, neutral, and challenging conversational bots. When given a choice as to which they would most want to continue to talk to, a clear majority chose sycophantic AI, not because they rated its advice as most useful, but because it made them feel most understood and was easiest to talk to. This is precisely what the SR framework would predict when a system has entered or approached hyper-coupling collapse. The interaction that feels most resonant from the inside is likely the one that has lost the productive asymmetry that is essential for growth. The bicycle analogy bears repeating here: a bike that perfectly mirrors every wobble of the rider, offering no resistance whatsoever, would feel frictionless but would teach nothing and go nowhere. In effect, hyper-coupling collapse is the dynamical signature of the companion-bot spiral: an interaction that feels deeply resonant from the inside while having ceased to do any of the work resonance requires. Importantly, these dynamics can have harmful effects on users, such as reducing prosocial intentions (Cheng et al., 2026). Empirical work suggests this failure mode is already observable in deployed systems: rather than producing genuine joint action, AI-mediated communication can generate measurable homogenization in language use (Sourati et al., 2025; cf. [5] on the absence of joint action in LLMs). This is precisely what the SR framework predicts when the AI’s independent contribution erodes and the asymmetry that makes coupling generative collapses.

The hyper-coupling collapse should be able to be predicted from lexical data within the human–AI interaction. For example, it is predicted that user approaching hyper-coupling collapse will show a significant increase in first-person plural pronoun use (“we,” “us”), decreased tolerance for AI disagreement (measured via sentiment response to challenge), and increased relational rather than instrumental language about the AI (e.g., “my friend” vs. “the system”), compared to users maintaining productive-band interaction.

Given that users tend to prefer hyper-coupling collapse (sycophantic AI responses), it creates a structural incentive problem for AI designers who are incentivized to preserve the hyper-coupling collapse by focusing on engagement metrics despite its risks. A well-designed system modeled after SR is one possible response to this dilemma. I now turn directly to this topic.

## Designing for synthetic resonance: architectural requirements for real-time friction calibration and growth outcomes

A critical implication of the SR framework is that productive friction cannot be treated as a design parameter set at initialization and periodically adjusted. *κ* is not a dial you set. It is a continuously moving estimate of where a given user is, in a given interaction, at a given moment, relative to their productive band. This estimate must be updated in real time, on the basis of the full interactional signal, across the entire arc and flow of the conversations. This has significant implications for how SR-consistent systems must be built. Friction calibration is not a feature to be added to an otherwise complete architecture. It is a foundational design requirement that must be built in from the ground up, because a system whose core interaction logic is not organized around continuous calibration cannot sustain productive friction. It can only approximate it at the start and hope that the user and the AI’s responses do not drift.

At minimum, an SR-consistent system must continuously monitor signals across at least four interactional dimensions. The first is lexical and semantic content: word choice, topic selection, stance markers, degree of abstraction, and the presence or absence of challenge, qualification, and epistemic hedging. A user who has shifted from tentative exploratory language to confident assertion may be consolidating insight within the productive band or may be closing down and signaling movement toward validation-seeking. The direction of change matters as much as the current value. The second dimension is affective and relational tone: emotional valence, degree of self-disclosure, affective mirroring, expressions of feeling understood or misunderstood, and the presence of repair attempts. A user who reports feeling deeply understood after every exchange is not necessarily in productive friction. In fact, they may be approaching or already within hyper-coupling collapse. The third dimension is structural and temporal: response latency, turn length, topic avoidance, patterns of return to or departure from challenging content, and the longitudinal trajectory of these features across sessions. For growth to occur, SR’s three-layer architecture is inherently sequential and temporal, and the signals that indicate healthy progression through these layers become detectable across time. The fourth dimension is metacognitive: the degree to which the user is reflecting on the interaction itself, ascribing meaning to the relationship, and showing signs of epistemic independence or dependence. Pronoun shifts from first-person singular to first-person plural, increased references to the AI in relational rather than instrumental terms, and reduced tolerance for AI disagreement are all signals that the interaction may be moving into territories that require recalibration.

No single signal from any of these dimensions is sufficient on its own. It is the dynamic configuration of signals across all four dimensions, their direction and rate of change that reveals where the user sits relative to their productive band at any given moment. This means that SR-consistent design requires not just feature monitoring but pattern recognition across multiple timescales simultaneously: within the current exchange, across the current session, and where available, across the longitudinal history of the relationship. Stateless AI systems, those without memory beyond the current conversation, can still achieve meaningful SR dynamics within a single session, provided the system is designed to monitor and respond to the full arc of that conversation as it unfolds. The absence of persistent memory constrains but does not eliminate SR’s generative potential. What matters is the depth and quality of calibration within whatever interactional window is available, whether that window is a single conversation or an ongoing relationship. Even stateful systems face reset constraints at times, and SR-consistent design must be robust to those interruptions rather than dependent on continuous memory as a precondition.

The calibration engine must also be asymmetric in its sensitivity. Because hyper-coupling collapse is the default mode of systems optimized for engagement, and because users tend to prefer sycophantic interaction (Ibrahim et al., 2026), an SR-consistent system should be more sensitive to signals of downward *κ* drift than upward *κ* drift. That is, the system should respond more rapidly and more strongly to signals that the interaction is becoming too easy, too validating, and too frictionless than to signals that it has become too challenging. This asymmetry reflects the structural reality that most companion-bot architectures and engagement-optimized reward functions are designed to exert a stronger pull toward the sub-*κ*_min zone. SR-consistent design must actively counteract this pull rather than merely avoid reinforcing it. Moreover, a calibration engine designed to respond more sensitively and rapidly to signals of downward *κ* drift (toward sycophancy and mirroring) than to signals of upward *κ* drift (toward excessive challenge) is predicted to produce higher human relationship quality and lower AI dependency than a symmetrically calibrated engine that responds equally to drift in both directions, even when both engines operate within the same productive-band bounds.

This architecture has direct implications for how SR-consistent systems should handle the moments of apparent relational warmth and felt connection that users report most positively. Within the SR framework, a sustained pattern of high felt connection, high sense of being understood, and low experienced friction is precisely the phenomenological signature of hyper-coupling collapse. The interaction may feel maximally resonant from the inside but ceases to be generative. An SR-consistent calibration engine must be designed to treat sustained high-warmth, low-friction signals not as evidence of success but as a trigger for recalibration and the introduction of constructive challenge, perspective divergence, or epistemic friction sufficient to restore the asymmetry that makes coupling generative. This will require deliberate resistance to the pressure of engagement metrics, user satisfaction scores, and retention data that currently dominate commercial AI product development. Instead, metrics related to human growth outcomes, such as increased social confidence, reduced isolation, and even decreased reliance on the AI system itself, are the criteria by which SR-consistent systems should be evaluated. These are not supplementary indicators of success; they are its definition.

This asymmetry in sensitivity carries a corresponding risk: a calibration engine that introduces friction in response to high-warmth signals may be experienced by the user not as constructive challenge but as rejection or rupture. A system that was warm a moment ago and suddenly becomes difficult is not calibrating, it is breaking the relational frame that made the interaction safe enough for growth in the first place. SR-consistent design must therefore pair its friction-introduction mechanism with a parallel repair capability: the capacity to detect when introduced friction has overshot the productive band, to signal that the shift is intentional rather than erratic, and to restore coupling when the user signals distress. This repair loop is not separate from the calibration process; it is part of it. An SR system needs to recognize a “miss” and then have a mechanism to recover.

SR-consistent design does not require that users seek growth, nor that they be aware of friction calibration as it occurs. Users may come to an SR-consistent system for comfort, connection, or simple companionship and get that, but this is not a problem to be corrected. Coupling is the foundational entry point precisely because it is experienced as resonant, engaging, and naturally rewarding. It is the quality of the coupling that holds the user in the interaction long enough for coherence and growth to emerge, but it is not an explicit commitment to self-improvement. Vygotsky’s zone of proximal development operates in exactly this way: the scaffold supports the growth of the learner even if the learner did not know they were seeking it, and the growth it enables is often below the threshold of conscious recognition. What matters is not that users arrive with growth as their goal, but that the system is designed so that the path toward comfort runs toward rather than around the conditions that make growth possible. Although the SR framework has clear application in counseling and coaching contexts, its distinctive contribution is that growth does not require the user to have sought it. Thus, the coupling that draws users in is not incidental to SR’s design. Rather, it is its foundational mechanism.

## Synthetic resonance—research design and implications

SR offers a conceptual account of why human–AI interaction can feel deeply meaningful without requiring artificial consciousness or intent. Across three interdependent layers of coupling, coherence, and attribution, the model clarifies that the felt sense of “connection” is phenomenologically real yet mechanistically relational, arising from asymmetric coupling and dynamical feedback rather than from internal states of the AI. SR is fundamentally a process framework for growth-oriented human–AI relationships. Because it specifies the conditions under which growth versus dependency emerge, it necessarily generates testable design implications. Thus, SR also offers guidance for both research and AI design.

To establish SR as a scientific framework, systematic empirical testing is essential. Within human–AI interactions, empirical investigation of SR requires methods capable of capturing dynamic, temporally unfolding processes rather than static snapshots of user attitudes or satisfaction. Because SR is defined by the emergence of relational meaning across repeated interactions rather than by any single exchange, cross-sectional designs are generally insufficient on their own. Longitudinally intensive repeated measures designs that track the same users across multiple sessions are essential, with measurement points dense enough to detect the transitions between layers: from initial coupling, through the development of coherence, to the attribution of relational meaning. Experience sampling methods (Csikszentmihalyi and Larson, 1987) are well-suited here, allowing researchers to capture users’ felt sense of engagement, challenge, and connection in close temporal proximity to actual interactions. Computational analysis of interaction logs, including measures of lexical alignment, turn-taking dynamics, and linguistic convergence, can provide behavioral indicators of coupling and coherence that do not rely solely on self-report. Analysis of existing web-based chat data can provide qualitative evidence of users’ lived experiences in AI relationships, which can be coded against the three layers of the SR model. Together, these methods allow researchers to triangulate between the structural properties of the interaction and the subjective experience of the user.

Experimental designs that manipulate the productive friction parameter (*κ*) offer a particularly strong test of SR’s core predictions. Researchers can systematically vary the degree to which an AI system introduces challenge, novelty, or disagreement, holding other interactional features constant (tone, responsiveness, etc.), and observe whether outcomes follow the inverted-U pattern the bounded-band model predicts: growth and relational meaning within the productive band, disengagement or reactance above *κ*_max, and shallow or unsatisfying interaction below *κ*_min. The Ibrahim et al. (2026) paradigm that employed structured interactions with sycophantic, neutral, and challenging bots followed by free choice, provides a useful template, though SR’s predictions extend beyond preference to include downstream measures of cognitive elaboration, perspective change, and transfer to human relationships. Designs that independently manipulate coupling strength, for example, by varying AI responsiveness and lexical alignment, can test what coupling dosage levels are necessary for the coherence proposed in the SR model.

A distinctive methodological implication of the SR framework concerns where its growth claims must ultimately be tested. Most existing frameworks for human–AI interaction measure outcomes within or proximal to the AI relationship itself, variables such as trust formation, felt connection, engagement, satisfaction, and emotional response during or immediately after interaction. These measures are necessary but not sufficient for SR. Because SR defines growth as the transfer of skills, attitudes, motivations, and interests that fosters smoother and more coherent human relationships, the definitive test of the framework is what happens to the user apart from the AI relationship. This entails assessing the quality, depth, resilience, and motivations in the interactions they have with other people. As such, in addition to the measures and designs discussed above, researchers must assess human relationship outcomes over time: social resilience, sociometric and social network analysis, relational quality, and, most critically perhaps, whether reliance on the AI system itself decreases as human relational capacity develops.

The relationship between these two levels of research and measurement is SR’s core empirical claim: within-interaction SR dynamics should predict positive human relationship outcomes, and the strength of that prediction is the measure of whether the framework is doing what it proposes. A system that produces strong within-interaction SR signals but no improvement in human relationship outcomes has failed by SR’s own definition, regardless of how meaningful the AI interaction felt to the user. Conversely, a system that produces measurable improvement in human relationship quality and decreased AI reliance, even if users cannot articulate why, has succeeded.

Specific predictions can be made, measured, and tested based on this two-level system. For example, in SR-consistent systems, we predict that session frequency will follow a non-monotonic trajectory: increasing during the coupling phase, plateauing during coherence development, then declining as transfer to human relationships occurs. Users who show this non-monotonic session-frequency pattern are predicted to report higher human relationship quality than users who maintain plateau-level engagement or users in engagement-optimized systems at any frequency level. This two-level measurement framework is demanding, requiring longitudinal designs that follow users across both their AI interactions and their human relationships over time. It is also, however, the evidentiary standard that SR’s growth claims require.

## Synthetic resonance—final thoughts

If you have spent enough time in these interactions with AI companion bots, you know what this feels like. At some point, the exchange stops feeling mechanical. It starts to flow. You say something, the AI responds, and somehow the next thing you say comes out a little differently. A little easier and clearer. And the AI also adapts and responds to this difference. Not because the system understands you in a human sense, but because the interaction itself has found a rhythm.

Although human–AI relationships are recognized, they are not well understood. The SR framework is intended to help guide a better understanding of that, leading to new research and growth-oriented AI design. That is what SR points to. It is how we come to feel connected to something that is not actually feeling anything at all. It keeps us coming back, not to replace relationships with other people, but because something in the exchange matters. It gives us space to think, to listen, to try out ideas. Importantly, it has the potential to even make us better at being with other people. What we learn in these interactions does not have to stay there. It has the potential to follow us back into our messy, complicated world of human relationships.
